# Resting behavior of broilers reared with or without artificial brooders

**DOI:** 10.3389/fvets.2022.908196

**Published:** 2022-07-26

**Authors:** Sara Forslind, Carlos E. Hernandez, Anja B. Riber, Helena Wall, Harry J. Blokhuis

**Affiliations:** ^1^Department of Animal Environment and Health, Swedish University of Agricultural Sciences, Uppsala, Sweden; ^2^Department of Animal Nutrition and Management, Swedish University of Agricultural Sciences, Uppsala, Sweden; ^3^Department of Animal Science, Aarhus University, Tjele, Denmark

**Keywords:** broiler, resting behavior, artificial brooder, disturbance, sleep

## Abstract

Rest and sleep are important for the welfare of mammals and birds. A large part of the daily time budget of broiler chickens is taken up by resting behavior and the quality of resting is important. However, in intensive broiler production systems, disruptions of resting behaviors are common. These disruptions of resting behavior could be negative for the health and growth of the birds. This study investigated if artificial brooders that provide a delimited and darker resting place, away from active birds, reduce disruptions of resting behavior compared to a control situation without artificial brooders. Six pens of each treatment were used in the same building, keeping 60 chickens (Ross 308) per pen. The artificial brooders were removed at 21 days of age. Data on disturbances and duration of resting bouts and activity between resting bouts were collected on 20 and 34 days of age. Also, as an indicator of the quality of rest, the animals' cognitive performance was evaluated in a spatial learning test that was performed at 11 days of age. The results showed that birds housed in pens with access to brooders have longer resting bouts (260.7 ± 5.2 vs. 132.8 ± 5.3s, *p* < 0.001) and are less likely to be disturbed during resting by other individuals (0.15 vs. 0.48, *p* < 0.001). The effect of the artificial brooders on both the duration of resting bouts and the proportion of disturbances remained after the removal of the brooders at 21 days of age. The duration of activity between resting bouts was shorter if the resting bout was ended by a disturbance (9.98 ± 1.0 vs. 61.0 ± 2.4s, *p* < 0.001). Birds reared with brooders were more likely to solve the spatial learning task (0.5 vs. 0.27, *p* < 0.01), but those succeeding were not faster at solving it. Broilers may be exposed to disrupted rest due to the lack of a dedicated resting place separated from areas with high activity. Using artificial brooders reduces disturbances but does not eliminate them. Therefore, additional changes to the housing conditions or management will be needed to prevent disturbances.

## Introduction

Rest and sleep are vital for the welfare of mammals and birds but are rarely considered in broiler production systems. Rest can be defined as a period of inactivity without any maintenance behaviors occurring, whereas sleep is a specific state of inactivity with altered consciousness and reduced responsiveness (1). Sleep cannot be distinguished from rest if only using behavioral measurements. Instead, an electroencephalogram (EEG) measuring brain activity is needed. Suggested functions of sleep include tissue restoration and growth, energy conservation, neurobehavioral and neurocognitive performance, memory processing, learning, and waste clearance of the brain (1–3). A certain duration of undisturbed sleep is needed to acquire both deep sleep and Rapid Eye Movement (REM) sleep, which together serves the vital function of sleep (3). In addition to being a welfare problem, disturbance of sleep may also affect productivity in farm animals (e.g., less growth, increased sickness, and possibly death) and thus profitability (3, 4).

Under natural conditions, a mother hen would influence the chicks' behavior to have regular resting periods throughout the day. Periodic brooding of the hen induces regular resting periods (5), but it also provides a heated dark area to rest under. The darkness provided by the mother hen, as well as the natural daily rhythm of brooding (6), differ a lot from the light/dark schedules used in broiler production systems which often consist of a long continuous light period with one dark period (1–6 h) each day. Schwean-Lardner et al. (7) showed that the duration of dark periods in poultry production systems is an important factor affecting rest. Specifically, longer periods of darkness decrease the duration of resting periods during light hours. Disturbances occur to a higher extent during the light phase than during the dark period (8) for which reason it may be expected that having dark periods during the day could possibly reduce the prevalence of disturbances. Using artificial brooders, that provide a dark and warm shelter to rest under, could attract chickens and motivate them to rest even during the day.

Apart from the lighting schedule, rest and sleep may also be affected by the design of the housing. Open areas have previously been shown to be avoided by chickens and instead, chickens gather along walls to rest when kept in a barren environment (9). The provision of functional areas for active behaviors such as eating, drinking, and dustbathing that are structurally separated from areas for resting may support undisturbed resting. A possibility to achieve this is the provision of elevated resting places. Chickens have an innate motivation to rest in elevated places, such as branches, but broiler chickens rarely use perches (10) probably due to their heavy body weight. Another elevated structure used for broilers is platforms, which are seen to be used from an early age, at least from 6 days of age (11). However, although elevated platforms reduce disturbances of rest in broilers to some extent they are not the ultimate solution to prevent disturbances as disturbances still occur (8). Here, we focus on artificial brooders as a measure to reduce the risk of active birds disturbing resting birds.

Artificial brooders have previously been used to separate active chicks from resting chicks, with the aim of reducing feather pecking in layer pullets and hens (12, 13). Riber (14) argued that artificial brooders may improve behavioral synchronization, specifically for inactive behaviors. Sleep in birds is associated with species-specific behaviors and may be triggered by specific environmental releasers or innate behaviors (15). For broiler chickens, a broody hen can be such a trigger as the chicks seek shelter under the hen for resting. This does not differ from layer chicks, but older domestic fowl rest in elevated places. Thus, an artificial brooder could possibly work as a replacement for a broody hen, allowing sleep patterns that are more like natural sleep patterns for chickens.

A barren environment, high stocking densities, and large flocks are commonly used in broiler production and can result in disturbances of rest and sleep. Disrupted sleep could lead to sleep fragmentation, which may lead to changes in cognitive function, including poor memory and difficulties in concentration (16, 17). Tartar et al. (18) show that rats learned the location of a platform in a water maze, but for rats having fragmented sleep the distance of swimming was longer indicating poorer memory indicating that sleep fragmentation negatively impacts spatial learning. Therefore, a spatial learning task may be a good indicator of sleep fragmentation, although never previously investigated in chickens.

In the present study, the aim was to investigate how resting behavior, including disturbances of rest, in fast-growing broilers, is affected by providing artificial brooders. We hypothesized that artificial brooders will improve broilers' quality of rest and that this would result in better performance in a spatial learning task. We expected that the frequency of disturbances would increase with the bird's age as they take up more space, resulting in conditions that are more crowded.

## Materials and methods

### Animals and housing

**Figure 1:**
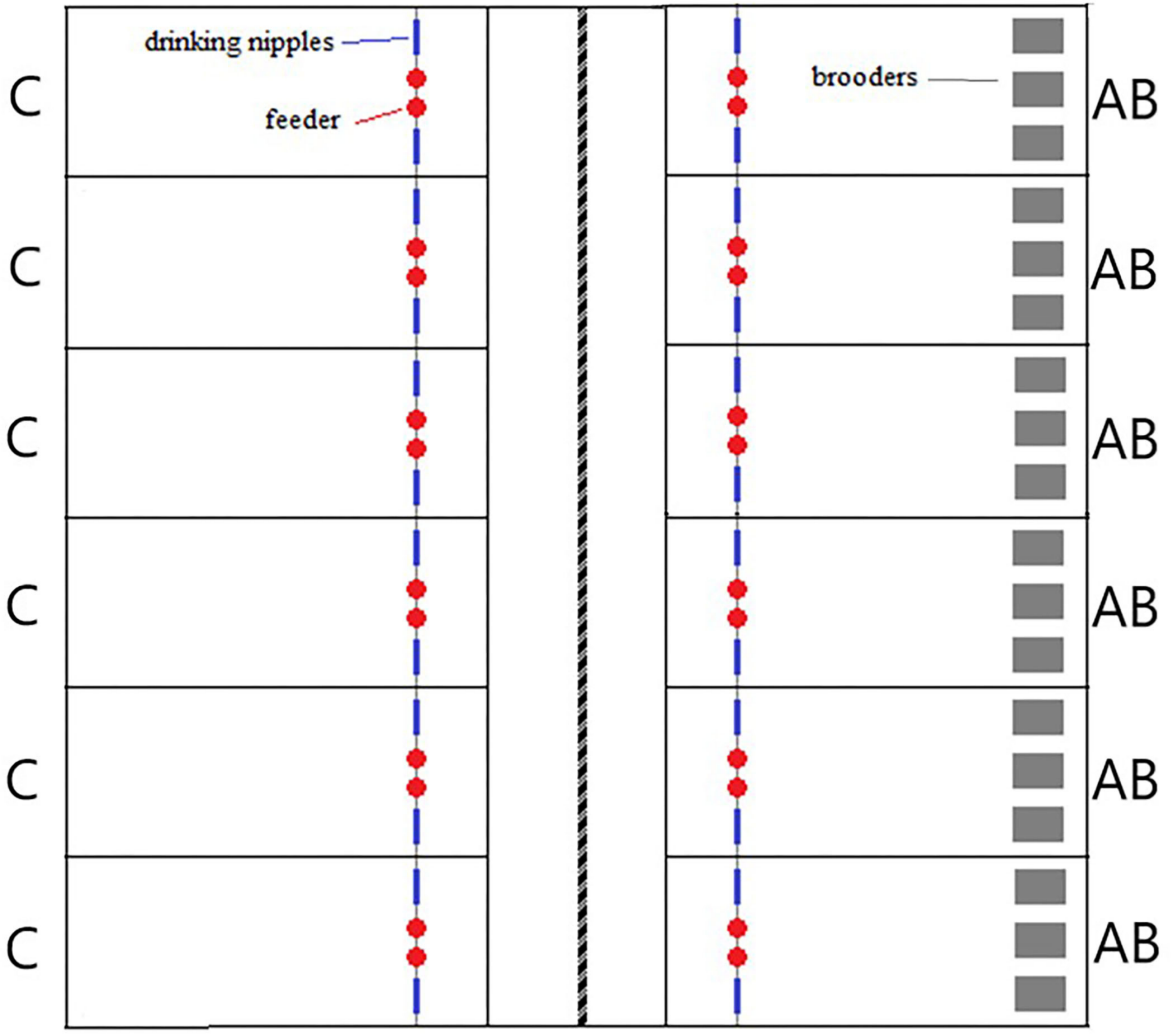
Layout of the poultry barn housing the experimental pens. Pens marked C are the control groups and those marked AB are the groups with artificial brooders.

This experiment was conducted at Lövsta Research Center, Swedish University of Agricultural Sciences, Uppsala, Sweden. In the building, one room was divided by a movable wall into two identical sections (6 m × 30 m) which were each equipped with six pens (12 pens in total) of 2 m × 3.5 m (7 m^2^; Figure 1). The pens were separated by 60 cm high wired fences and the floor was covered with a 4 cm layer of wood shavings.

A total of 720 Mixed-sex Ross 308 broilers were picked up as day-old from a commercial hatchery (Swehatch AB, Väderstad, SWE) and transported by car to the research facilities. Upon arrival, the chicks were randomly allocated into the pens, resulting in 60 chicks per pen. In one section, the ambient temperature was kept according to commercial practices with a starting temperature of 34°C and gradually decreased to reach 20°C at 21 days and to the end of the growing period. The other section kept an ambient temperature of 20°C throughout the entire growing period and in each of these pens, three artificial brooders (40 cm × 60 cm, vidaXL) were provided with a starting temperature of 34°C measured on the floor. The temperature of the artificial brooders was gradually decreased to reach 28°C at 21 days of age and the artificial brooders were then removed from the pens. The stocking density was kept at an expected 20 kg/m^2^ at slaughter age to give room for different resting place opportunities. Water was provided *ad libitum* by nipple drinkers (10 broilers/nipple) and feed was provided in round feeders (2 cm feeder space per bird). Birds were fed a recommended commercial grower diet *ad libitum* (feed company Lantmännen, SWE). At 1 day of age, the light schedule was programmed for 24L: 0D. Subsequently, the dark period was gradually increased to 6h on day 6 of age (18L: 6D) and maintained until the end of the experiment (the light was on 04:30–22:30). No daylight was provided. The light intensity was 27 lux at the animal level and 0–2 lux under the brooders. The study ended at 35 days of age, when the birds were slaughtered.

### Treatments

**Figure 2:**
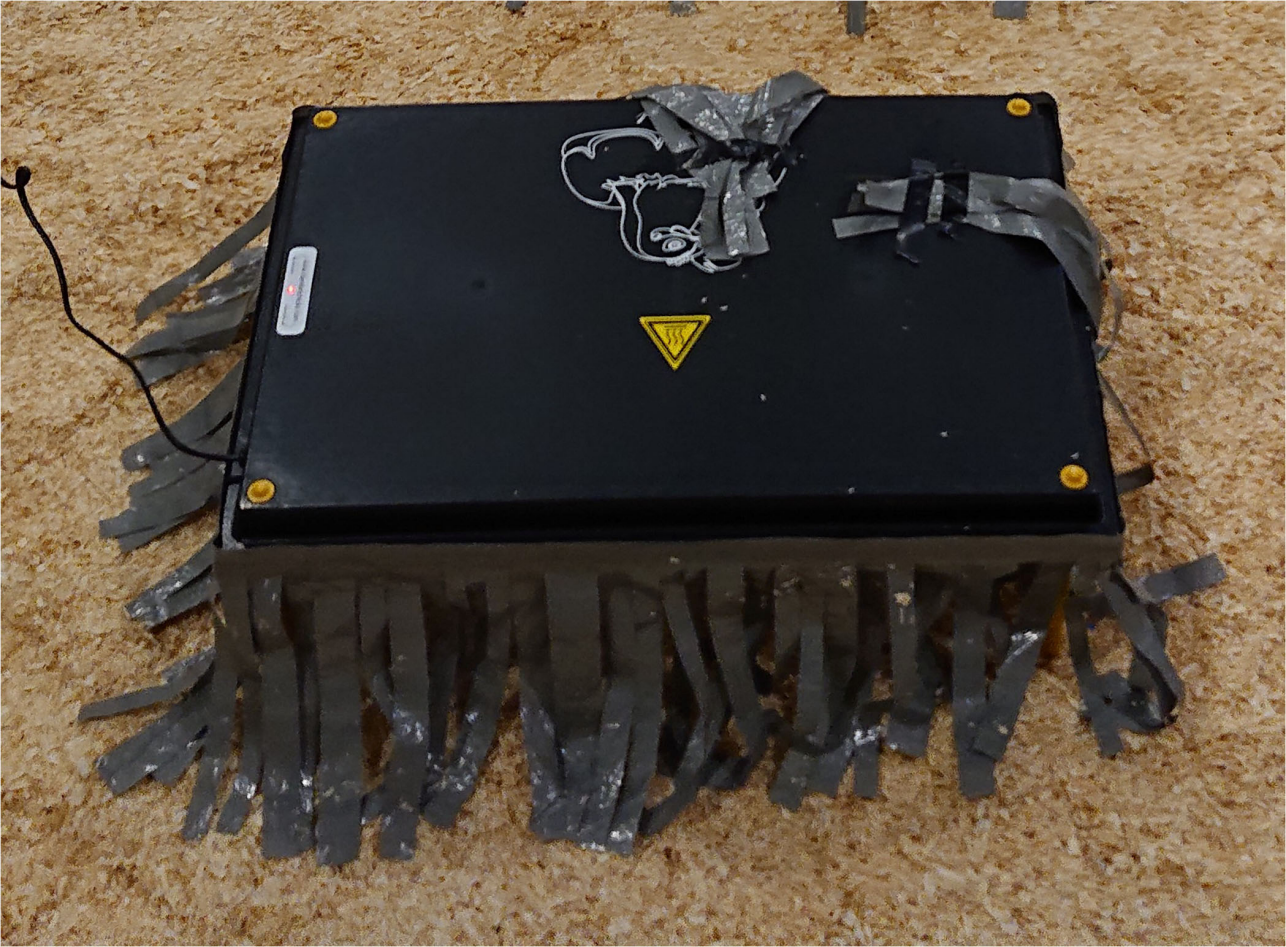
Artificial brooder with flaps of tarp. The height of the brooders was adjusted during the experiment.

Two treatments were used in this study, artificial brooders and control without brooders. In the treatment with brooders, each pen had three artificial brooders (40 cm × 60 cm) with the sides of the brooders covered with flaps of the tarp to make the area under the brooders dark (Figure 2). The brooders were removed when they were 21 days when all chicks no longer could fit under them and the heat provided no longer was necessary.

### Data collection

Cameras (Sony SNC-CH120) were mounted on the ceiling, facing directly downwards, each camera covering two whole pens but with “dead spots” under the brooders. Small cameras (GoPro Hero 7 White) were used to record under the brooders and were only present during recording (a wire mesh cage for the camera was always present under each brooder). Data on the use of the brooders were collected using scan sampling four times a day on days 6, 13, and 20. Data on resting behavior were collected on days 20 and 34 of age from the video recordings using focal animal sampling. Each pen was observed two times per observation day (days 20 and 34), at morning from 06:00 to 08:00h and in the evening from 20:00 to 22:00h. The videos showed that the pen was divided into nine imaginary squares of equal size. A random number was given for each new observation and an individual in that square was followed. A total of 10 individuals per pen and observation period (morning and evening of days 20 and 34) were observed. In addition, using videos recorded under the brooders, 10 individuals per pen were also followed. Focal animals were chosen once individual birds started to rest (defined as lying with a leg to the side or sitting with the legs under the body while not engaging in any other activities), chosen in a randomized square of the pen (randomization through a given list of numbers between one and nine). The focal animals were followed during a complete resting bout as well as the following activity, defined as all behaviors that do not fit in our definition of rest, until the start of the next resting bout. Chickens that rested under brooders, but left the brooder when becoming active, were followed using the cameras above the pen. The length (in s) of each resting bout and the duration of activity between resting bouts as well as the occurrence of disturbances (defined as physical disturbances by other individuals, causing the focal animal to change position or become active) were registered. In addition, it was registered whether the position of the focal animal in the pen was (1) under the brooder (only in the treatment with brooders), (2) close to a wall, defined as being within one bird length from a wall, or (3) elsewhere in the pen (open areas). One observer collected all data from the videos to avoid the confounding effects of several observers.

**Figure 3:**
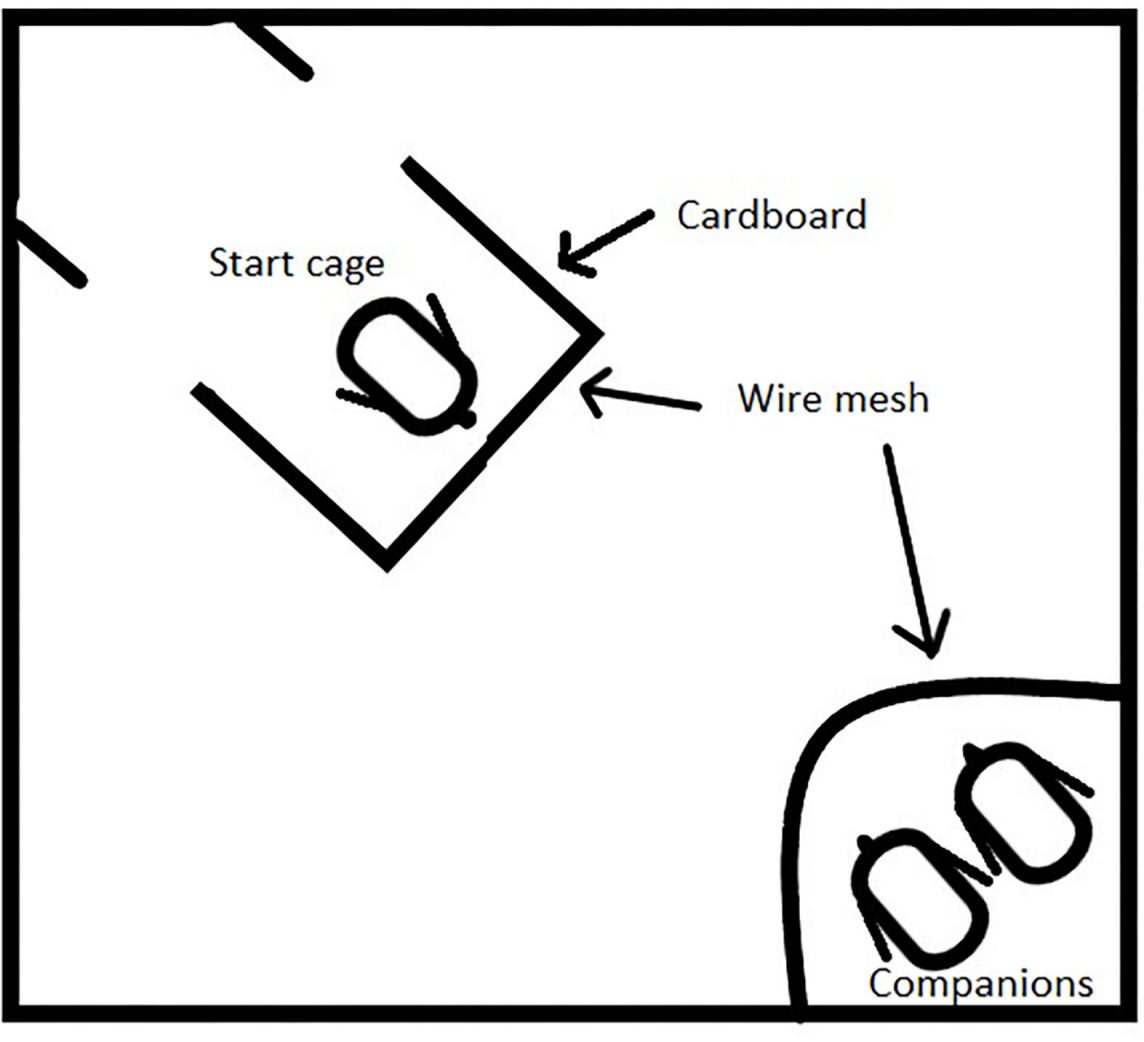
Spatial learning arena. A companion cage with two companions were put in one corner. The starting box in the corner diagonally had wire mesh toward the companions and two openings behind the chick's starting position.

A cognitive test was performed to evaluate spatial learning capacity, adapted from the study by Freire et al. (19). At 11 days of age, five birds from each pen (30 per treatment) were randomly chosen. Chicks were carried, in a box with companions, to a separate room and given 10 min to acclimatize, with the companions, to the environment. An 80 cm × 80 cm white box with 60 cm high white panels was used as an arena (Figure 3). A wire mesh cage of 15 cm × 15 cm was placed in one corner, where two companion birds were placed and provided with feed and dried mealworms. The companions came from the same pen as the bird to be tested and were not used for testing themselves. In the corner diagonally from the cage with companion birds, a three-sided cage was placed 10 cm from the wall. The cage had two sides of cardboard and one wire mesh side facing the other cage, the back was open to allow the chick to leave the cage and get closer to the companions. The distance between the cages was 70 cm. The test started when a chick was placed in the starting cage and ended when the chick was one bird length from the companion cage or after a maximum of 10 min if the chick was unsuccessful to reach the companions. The test was recorded using GoPro Hero 7 cameras to avoid human interference and videos were later analyzed. The latency for the chick to leave the starting cage (passing one of the cardboard edges) and the latency to reach one bird length of the cage of the companion birds were noted. A shorter latency implies a better understanding of the spatial environment. One observer conducted the experiment and collected all data from the videos to avoid the confounding effects of several observers.

### Statistical analysis

Statistical analyses were performed using R (R version 4.1.3, 20). The significance level used in the study was 0.05.

An ANOVA test was used to compare the durations of resting bouts and activity between resting bouts between the treatment groups. The explanatory factors used in this model were treatment, position in the pen, age, and period of the day and the random factor used was the pen. The interactions between the explanatory factors were also included in the initial model, but they were removed when not statistically significant. After a logarithmic transformation of the duration data, the data adhered to normal distribution and homogeneity of variances. *Post hoc* comparisons of significant factors were performed using Tukey's HSD test. Results are reported as means ± SE.

A Chi-squared test was used to test the occurrence of disturbances between treatments and positions. As the brooders were removed between the two observation periods, the test was done separately for each age. The explanatory factors used in this model were treatment and position in the pen. Results are reported as means.

A *t*-test was used to investigate the effect of disturbances on the durations of resting bouts and activity between resting bouts. All original data from both treatments, age, and period of day were used. After a logarithmic transformation of the duration data, the data adhered to normal distribution and homogeneity of variances. Results are reported as means ± SE.

A Chi-squared test was used to test the proportion of birds solving the spatial learning task between treatments. Thereafter, a Kruskal–Wallis test was used to test for latencies between treatments. Data did not adhere to normal distribution. Results are reported as means.

### Ethical statement

The study was approved by the Animal Research Ethics Committee in Uppsala (Dnr 5.8.18-17765 2018).

## Results

### Usage of brooders

Chicks reared with brooders were seen under the brooders at all ages (Table 1).

**Table 1:** Proportion of chicks being under the brooders during observation four times a day at ages 6, 13 and 20.

**Age (days)**	**Time of day**	**Proportion of chicks under the brooders**
6	06:00	0.27
6	08:00	0.28
6	20:00	0.26
6	22:00	0.28
13	06:00	0.25
13	08:00	0.24
13	20:00	0.24
13	22:00	0.22
20	06:00	0.16
20	08:00	0.14
20	20:00	0.18
20	22:00	0.19

### Duration of resting bouts

There were no significant interactions between the explanatory factors treatment, age or period of the day for duration of resting bouts. A treatment effect was found on the duration of resting bouts (*df* = 1, *F* = 375.0, *p* < 0.001) where the treatment with brooders had longer resting bouts than the control treatment (260.7 ± 5.2s vs. 132.8 ± 5.3s). The position in the pen mattered for duration of resting bouts (*df* = 2, *F* = 29.6, *p* < 0.001). Resting bouts taking place under the artificial brooder (329.4 ± 8.4s) were longer than in open areas (176.5 ± 6.3s, Tukey's test *p* < 0.001) and near walls (182.7 ± 6.0s, Tukey's test *p* < 0.001). Duration of resting bouts taking place in open areas did not differ from resting bouts taking place near walls (*p* = 0.254). The resting bouts were longer in the evening (*df* = 1, *F* = 33.5, *p* < 0.001) than in the morning (Evening vs. Morning: 229.1 ± 6.4s vs. 190.0 ± 6.4s). The resting bouts were longer for older birds (*df* = 1, *F* = 5.3, *p* = 0.02) than younger birds (20 vs. 34 days of age: 202.3 ± 6.4s vs. 216.8 ± 6.5s).

### Duration of activity between resting bouts

There were no significant interactions between the explanatory factors treatment, age or period of the day for duration of activity between resting bouts. There was a treatment effect of the duration of activity between resting bouts (*df* = 1, *F* = 21.85, *p* < 0.001) where the treatment with brooders had longer activity than the control treatment (49.2 ± 2.4s vs. 40.0 ± 3.3s). The position in the pen while resting prior to becoming active also affected the duration of activity between resting bouts (*df* = 2, *F* = 6.5, *p* < 0.01) where birds resting in open areas (37.7 ± 2.7s) were active for a shorter duration than birds resting under the artificial brooders (52.2 ± 4.2s, Tukey's test *p* = 0.013) and near walls (50.0 ± 3.5s, Tukey's test *p* = 0.006). Duration of activity after resting near walls did not differ from resting under brooders (Tukey's test *p* = 0.935).

### Proportion of resting bouts disturbed

There was a difference in the proportion of disturbances between the treatments both at 20 days of age (*df* = 1, χ^2^ = 37.8, *p* < 0.001) and at 34 days of age (*df* = 1, χ^2^ = 12.2, *p* < 0.001) with a lower proportion of resting bouts being disturbed in the treatment with brooders (0.15 and 0.25 disturbed) than in the control treatment (0.48 and 0.42 disturbed). There were no differences between positions within the treatments. At 20 days of age, the proportion disturbed in the treatment with brooders was 0.08 under the brooder, 0.23 in open areas and 0.13 near walls. At 34 days of age, the proportion disturbed in the treatment with brooders was 0.23 in open areas and 0.27 near walls. In the control treatment, the proportion disturbed in open areas was 0.55 and near walls 0.42 at 20 days of age. At 34 days of age, the proportion of disturbed in open areas was 0.53 and near walls 0.42 in the control treatment.

### Influence of disturbances on durations of resting bouts and activity between resting bouts

**Figure 4:**
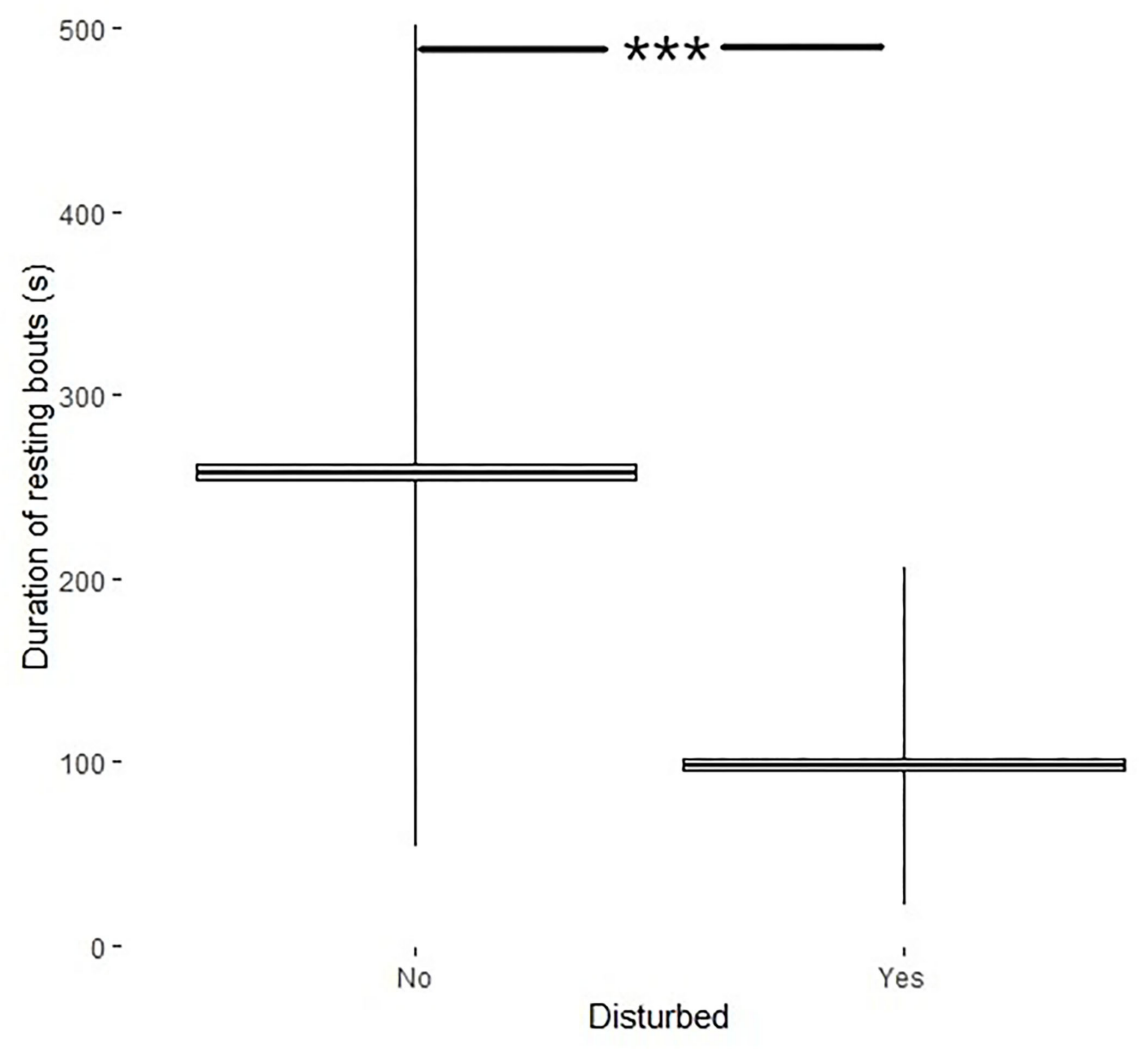
Duration of resting bouts (s, min–max + mean ± SE) if the bird was disturbed (yes) or not (no). ***Indicates significance level.

The analyses of influence of disturbances on length of resting bouts and length of periods of activity between resting bouts were performed on pooled data. Disturbances affected the duration of resting bouts (*df* = 290, *t* = 23, *p* < 0.001) where resting bouts of disturbed birds were shorter (98.4 ± 3.4s) than the resting bouts if no disturbance occurred (257.9 ± 4.7s) (Figure 4).

**Figure 5:**
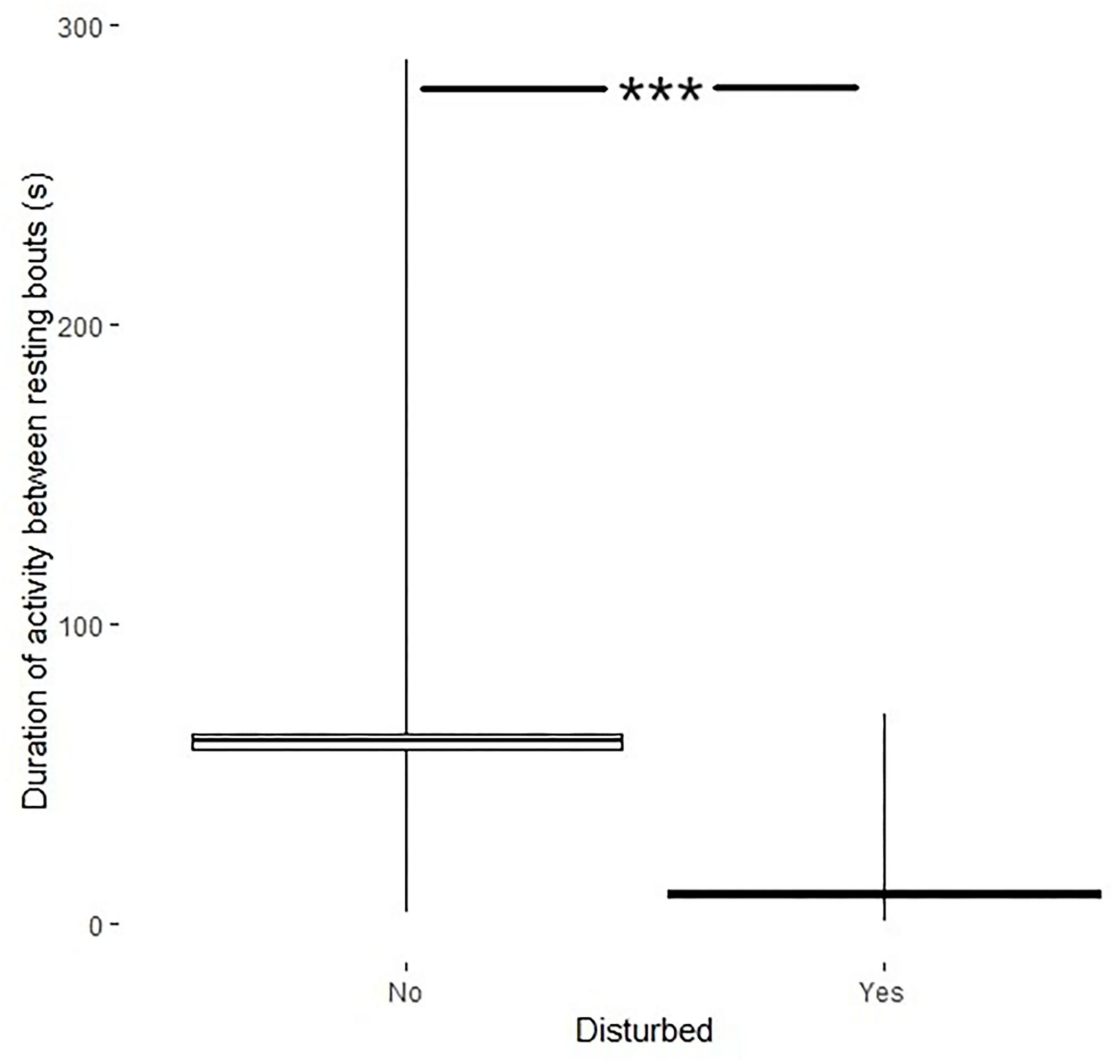
Duration of activity between resting bouts (s, min–max + mean ± SE) if the bird was disturbed (yes) or not (no). ***Indicates significance level.

Disturbances also affected the activity between resting bouts (*df* = 287, *t* = 25.6, *p* < 0.001) where the duration of activity was shorter after a disturbance (9.98 ± 1.0s) than the duration of activity when no disturbance had occurred (61.0 ± 2.4s; Figure 5).

### Spatial learning

The spatial learning task showed a difference between treatments in the proportion of birds successfully solving the task, i.e., leaving the start cage (*df* = 1, χ^2^ = 10.2, *p* < 0.01) where more birds from the treatment with brooders left the start cage (Table 2). No differences between treatments were found in latencies for the birds to leave the start cage (*df* = 1, Kruskal-Wallis chi-squared = 0.20, *p* = 0.65) or reach the companion cage (*df* = 1, Kruskal–Wallis chi-squared = 2.03, *p* = 0.15), for birds that left the start cage (Table 2).

**Table 2:** Proportion of chickens leaving the start cage in a spatial learning task and latencies (s) to either leave the start cage or reach the companion cage.

**Treatment**	**Proportion of chickens leaving start cage**	**Latency to leave start cage**	**Latency to reach companion cage**
Brooders	0.5a	197.5 ± 36.5a	227.4 ± 36.8a
Control	0.27b	203.1 ± 24.6a	229.6 ± 24.7a

Different letters within parameter indicate significant treatment differences (p ≤ 0.05).

## Discussion

This study shows in general that there are frequent physical disturbances causing individual chickens to end resting bouts throughout the day. Disturbances were common in both treatments. Similarly, in a study by Yngvesson et al. (10), 53% of the 45-day old focal birds were disturbed during resting at daytime. Also, Forslind et al. (8) found that a high proportion of resting bouts ended due to the chickens being disturbed by other individuals, both during night and day, again suggesting that physical disturbances are common. Artificial brooders have been suggested to provide a separate resting place where chicks can go to rest, away from active chicks (13). Since the number of disturbances was lower under the brooders compared to the rest of the pen and compared to the control, they seem to some extent to fulfill the suggested hypothesis of being a separate resting place. Similarly, elevated platforms seem to provide a resting place, away from active individuals, as it has been shown that elevated platforms reduce the proportion of disturbances among birds resting on them, when observed at days 20 and 34 (8).

As both treatments had the same conditions after 21 days of age due to the removal of brooders, one could expect to find less differences in the behavior of the chickens at 34 days of age. However, the brooder treatment caused a lower number of disturbances even after the removal of the artificial brooders compared to the control treatment. Also, the duration of resting bouts were longer in the brooder treatment than in the control treatment even after the removal of the brooders. This means that we see a long term effect of using the brooders early in the rearing, which has previously been shown on other behavioral aspects in layers, e.g., reduction of feather pecking and fear (e.g., 21, 22). The mechanisms of the long-term effect seen in resting behavior need to be further studied.

A certain period of undisturbed sleep is necessary to reach specific sleep stages like Rapid Eye Movement (REM) and since poultry also show REM-like sleeping patterns (20), a longer period of undisturbed sleep is likely to be important. In the present study, the resting bouts were longer, both if the birds were disturbed or not, in comparison with the resting bouts observed in Forslind et al. (8) where broilers were kept at a stocking density of 40 kg/m^2^ with access to elevated platforms or at a stocking density of 34 kg/m^2^ without access to elevated platforms. There are several differences between the studies, but one major difference is the stocking density. In the present study, the stocking density was kept at 20kg/m^2^ whereas in Forslind et al. (8), the stocking density was kept as in commercial practice for Danish conditions, i.e. 40 kg/m^2^ (or 34 kg/m^2^ for the low-density treatment). The expectation was that with additional space birds can move about with less physical contact, reducing the need for birds to run over each other. Indeed, Dawkins et al. (21) showed that the disturbances increased with stocking densities where differences were found between a stocking density of 30 and 42 kg/m^2^ or higher. Another reason for choosing a low stocking density was for the birds to all fit under the brooders, until 3 weeks of age. To gain this with a higher stocking density would imply covering more of the pen in brooders, which would reduce the opportunities for chicks to choose a resting place away from the brooders.

To get an impression of the motivation to rest, and thereby of the impact of disturbances, the duration of activity between two resting bouts was observed. When a resting bout was followed by only a short phase of activity, the chicken can be considered to have high motivation to continue resting. In the current study, the average time the chicks spent active was 40–50 s. In a study with elevated platforms, where resting bouts were found to be shorter and the proportion of resting bouts being disturbed to be higher, the activity between resting bouts was very short, around 10–15 s (8). This might be interpreted as that either brooders or the lower stocking density or the combination, gave the chickens a better quality of rest as the motivation to continue resting after becoming active was lower.

During the spatial learning task, twice as many birds with access to artificial brooders were successful in solving the task and leaving the start cage than birds reared without brooders. As our assumption was that birds that sleep better also have better cognitive skills, the lower proportions of disturbances and longer resting bouts within the treatment with brooders could have affected the outcome of the test. That would be supported by Tartar et al. (18) who showed that rats perform better in a water maze if not exposed to fragmented sleep. Also, Johnsson et al. (22) showed that sleep-deprived magpies performed worse in a reversal learning task and had lower motivation to complete the task. Sleep fragmentation could possibly be a reason for the results, as sleep fragmentation affect learning and memory (16), but it is unknown in the current study whether the chicks were experiencing sleep fragmentation. However, there could also be an effect of the occlusion by the brooders in the pen, since birds reared with brooders may have experienced situations where walking around the brooders was needed in order to find companions. Freire et al. (19) saw that chicks reared with the option to walk out of sight from an imprinted mother also performed better in a spatial learning task. The spatial learning task has limitations (e.g., it is only one test, not all individuals from each treatment were tested and it is unknown if the tested individuals from the brooder treatment used the brooders), thus preferably another spatial learning task should be done to confirm the results in future studies.

A main reason for the high frequency of disturbances in both treatments is likely the lack of behavioral synchronization. When resting, chickens seek each other's company and when not synchronized in behavior they continuously enter and leave resting groups and areas, disturbing resting birds. Riber (14) showed that artificial brooders could somewhat act as a cue for social synchronization, specifically for inactive phases (23–25). Additional measures to better synchronize behavioral patterns would be needed. This could potentially be intermittent lighting programs, which could act as a signal for the chicks to initiate resting phases and therefore possibly synchronize resting behavior in the flock.

## Conclusion

In this study, the frequency of disturbances and duration of resting bouts showed that individuals experience difficulties in achieving undisturbed rest. The introduction of artificial brooders provided an opportunity for somewhat longer and less fragmented rest. However, disrupted rest was common in all situations suggesting that more measures than adding an artificial brooder are needed to further reduce disturbances. Increased synchronization of behavioral patterns could possibly be such a measure that further reduces disturbance, but more research is needed to determine how to induce it in broiler flocks and to evaluate its potential effects on quality of sleep.

## Data availability statement

The raw data supporting the conclusions of this article will be made available by the authors, without undue reservation.

## Ethics statement

The animal study was reviewed and approved by Uppsala djurförsöksetiska nämnd, Jordbruksverket, Sverige.

## Author contributions

SF, CH, AR, HW, and HB contributed in the planning of the project, both teoretical and practical. SF conducted the experiment, data collection, analysis, and wrote the manuscript with input from CH, AR, HW, and HB. All authors contributed to the article and approved the submitted version.

## Funding

The research described in this paper was funded by the Swedish Research Council Formas and the Swedish Board of Agriculture.

## Conflict of interest

The authors declare that the research was conducted in the absence of any commercial or financial relationships that could be construed as a potential conflict of interest.

## Publisher's note

All claims expressed in this article are solely those of the authors and do not necessarily represent those of their affiliated organizations, or those of the publisher, the editors and the reviewers. Any product that may be evaluated in this article, or claim that may be made by its manufacturer, is not guaranteed or endorsed by the publisher.
